# Supplementary material for: A Bayesian network for modelling the Lady tasting tea experiment
Source: PLoS One. 2024 Jul 25;19(7):e0307866. doi: 10.1371/journal.pone.0307866 (PMC11271908; doi:10.1371/journal.pone.0307866)
Supplement: Attachment — Submitted filename: Response to Reviewers_Xie_BNmodel.docx [file pone.0307866.s002.docx]

7 June, 2024

To: Gonzalo A. Ruz, Ph.D.
 Academic Editor
 PLOS ONE

Dear Dr. Gonzalo A. Ruz,

Thank you for the opportunity of revision of our manuscript. We really appreciate the time and effort you and the reviewers committed in reviewing our manuscript and providing the valuable feedback for the revision. We have carefully considered all the comments and have made the necessary revisions to improve the quality of our work. Below, we provide a detailed, point-by-point response to each comment. The reviewers' comments are included in italics, followed by our responses in regular font.

**Reviewer #1:**

**Comment 1:** *“I believe the authors have done an excellent job in describing an empirical case of the lady tasting tea experiment and exemplifying their use of the BN model in this paper. Figures 1-5 and the Table 1 are clear and well presented. The paper is well organized and easy to read/follow. I have no challenges or questions in regard to the application as highlighted in this paper.*”

**Response:** We are sincerely appreciate your positive feedback and delighted to hear that you found our manuscript to be well-organized, clear, and easy to follow. We are pleased that our description of the empirical case and the use of the BN model met your expectations. Your recognition of the clarity of Figures 1-5 and Table 1 is very encouraging. Thank you for your kind words, which motivate us to continue our research with the same rigor and clarity.

**Comment 2:** “*The authors may suggest follow-on research to expand upon their ‘hopeful’ acceptance/adoption of the method as another case of limitations of Fisher’s testing of significance. Also, they should call out limitations for this BN modelling approach.*”

**Response:** Change has been made by adding a few sentences at the end of Discussion section to suggest directions for follow-on research.

**Comment 3:** “*Suggested Edits*

*INTRODUCTION*

*Needs reference:*

*In 2001, David Salsburg published his book ‘The Lady Tasting Tea: How*

*Statistics Revolutionized Science in the Twentieth Century,’ which was well-received by a*

*wide range of reader*

*Lines 81-82 –*

*Drop can--“can fully characterises and represents the probabilistic and statistical properties of the lady tasting tea experiment.”*

*METHOD AND RESULTS*

*Line 85 – change “maybe” to “may be”*

*DISCUSSION*

*Line 153 – “Therefore” not needed*

*Lines 153-154 -- Drop “that”:*

*Lines 156-158 “Hopefully this BN model can…” – Perhaps state this more positively vs hedging—“This BN model can…”*

*“Therefore, this article has presented a BN that modelling the lady tasting tea experiment which allows us to perform…”*

*to*

*“Therefore, this article has presented a BN modelling the lady tasting tea experiment which allows us to perform…”*

*Line 162 – comma after paradigm*

*Line 167 – Change to “were served”*

*Line 171 – Change to “a different prior distribution”, OR “depending on different prior distributions”* ”

**Response:** Thank you for your thorough editing check of our manuscript. We have carefully examined all your suggestions and have incorporated them into the revised version.

**Comment 4:** “*Lines 206-207—Bayesian network model in supporting information—I was not able to access this BN model that is supposed to be in the supporting information to check/assess it.*”

**Response:** We have indeed submitted the BN model file as part of the supporting information when the manuscript was first submitted. You may check with the Editor to request the submitted BN model for assessment. Otherwise, we are more than happy to provide the BN to you directly upon receiving permission of the journal Editor.

**Reviewer #2:**

**Comment 1:** “*The article seems to have been written somewhat carelessly. It would be good to organize the article and review the notations used (in detail). It is not clear how reproducible and reusable the experiment is. It would be helpful to provide more details about how the program works.*”

**Response:** We thank this reviewer for his/her time and effort in reviewing our manuscript and providing the valuable feedback. Since the feedback was generally broad, we have done our best to respond in a more specific manner and hope our responses meet reviewer’s expectations.

The structure and style of our manuscript may not follow exactly the common pattern of a research project journal paper because the very purpose of this study is to provide a novel Bayesian solution to the classic Frequentist hypothesis testing question based on the well-known the Lady Tasting Tea experiment that was first introduced by R. A. Fisher some 100 years ago. In the writing of the manuscript, we implicitly assumed that the target readers were familiar with major statistical paradigms (e.g., frequentist and Bayesian approaches) and Bayesian network (BN). This assumption may have been unrealistic. To address the reviewer’s concern, we have therefore added a few paragraphs in the Method and Results section to give a brief introduction to the theoretical foundation of Bayesian network and how BN works for statistical analysis.

We have employed standard mathematical and statistical notations consistent with year-one undergraduate-level textbooks. However, the concepts such as prior and posterior probabilities may be new to readers who are not familiar with Bayesian statistics. The distinction between different probability definitions (e.g., relative frequency definition versus propensity definition) can be both philosophical and challenging to appreciate.

The analyses conducted using the BN model and the results presented in the manuscript can be fully reproduced. We have indeed submitted the BN model file as part of the supporting information when the manuscript was first submitted. You may check with the Editor to request the submitted BN model for assessment. Otherwise, we are more than happy to provide the BN to you directly upon receiving permission of the journal Editor.

All the changes made to the manuscript can be identified clearly in the marked-up copy of the revised version of our manuscript.

Thank you again for your time and effort in handling our manuscript.

Sincerely,

Gang Xie

Charles Sturt University, Australia
